# Endotoxemia and its association with immune and coagulopathy responses in severe community-acquired pneumonia and COVID-19

**DOI:** 10.1186/s40635-026-00863-y

**Published:** 2026-03-09

**Authors:** Mathieu Blot, Amadou-Khalilou Sow, David Masson, Maxime Nguyen, Marine Jacquier, Jean-Paul Pais de Barros, Thibault Sixt, Maxime Luu, Pierre-Emmanuel Charles, Lionel Piroth, Jean Pierre Quenot, Christine Binquet, Lymphonie Study Group, Jean-Pierre Quenot, Pierre-Emmanuel Charles, Marine Jacquier, Pascal Andreu, Marie Labruyère, Jean-Baptiste Roudaut, Sébastien Prin, Jose Pineda, Alicia Taha, Philippe Bonniaud, Marjolaine Georges, Guillaume Beltramo, Bernard Bonnotte, Hervé Devilliers, Jérémy Barben, Lionel Piroth, Thibaut Sixt, Julien Guy, Christine Binquet, Abderrahmane Bourredjem, Amadou-Khalilou Sow, Florine Cattin, Marc Bardou, Maxime Luu, Ines Ben Ghezala, Karen Sagorny, Julie Ferrandes, Joao Pais-De-Barros, Hélène Choubley, Victoria Bergas, Julie Maréchal, Laure Stiel, Sylvie Nguyen, Mathieu Blot

**Affiliations:** 1 Université Bourgogne Europe, CHU Dijon Bourgogne, département de Maladies Infectieuses, 21000 Dijon, France; 2 Center for Translational and Molecular Medicine, INSERM U.M.R. 1231, Lipness Team and LabEx LipSTIC, University of Burgundy, 21 000 Dijon, France; 3 Université Bourgogne Europe, CHU Dijon Bourgogne, Centre d’Investigation Clinique, Module épidémiologie clinique, INSERM, CIC1432, 21000 Dijon, France; 4 Université Bourgogne Europe, CHU Dijon Bourgogne, laboratoire de Biochimie, 21000 Dijon, France; 5 Université Bourgogne Europe, CHU Dijon Bourgogne, Département d’anesthésie et réanimation, 21000 Dijon, France; 6 Université Bourgogne Europe, CHU Dijon Bourgogne, Département de médecine intensive et réanimation, 21000 Dijon, France; 7 Plateforme de lipidomique, Université de Bourgogne, 21 000 Dijon, France; 8 Université Bourgogne Europe, CHU Dijon Bourgogne, Centre d’Investigation Clinique Université Bourgogne Europe, CHU Dijon Bourgogne, Centre d’Investigation Clinique, Module Plurithématique, INSERM, CIC1432, 21 000 Dijon, France

**Keywords:** Community-acquired pneumonia, Endotoxemia, Lipopolysaccharide, Immune response, Coagulopathy, Thrombosis, Cardiovascular event, Mortality

## Abstract

**Background:**

Acute community-acquired pneumonia (CAP) is a leading cause of infection-related mortality worldwide. Endotoxemia, characterized by elevated plasma lipopolysaccharide (LPS), is a key driver of inflammation and thrombosis in Gram-negative sepsis and has been suggested to occur in severe pneumonia, irrespective of etiology. However, current immunoassays for LPS quantification lack sensitivity and specificity. We aimed to quantify plasma LPS in severe CAP patients, including COVID-19, using a validated mass spectrometry method, and to explore associations with immune activation, coagulation, gut translocation, and clinical outcomes.

**Methods:**

In this prospective ancillary study of the LYMPHONIE cohort, we included 34 non-COVID-19 severe CAP (sCAP), 34 severe COVID-19 (sCOVID-19) and 34 matched healthy volunteers. Plasma LPS was measured by LC–MS/MS detecting 3-hydroxy fatty acids of lipid A. Clinical data, immune biomarkers, coagulation biomarkers, and gut injury markers were measured.

**Results:**

Unexpectedly, median plasma LPS concentrations were significantly lower in sCAP patients (724 pmol/ml in sCAP; 750 pmol/ml in sCOVID-19) compared to healthy volunteers (1009 pmol/ml, *p* < 0.001). LPS levels did not correlate with severity scores or mortality. Low positive correlations were observed between LPS and markers of endothelial activation (sVCAM-1) and coagulation (D-dimer). However, patients with high LPS showed no increased risk of thrombotic or cardiovascular events.

**Conclusions:**

Using a highly specific LC–MS/MS method, we found no evidence of increased circulating LPS in severe pneumonia patients, challenging the hypothesis of gut-derived endotoxemia as a major contributor to systemic inflammation in severe CAP, including COVID-19.

***Take-home message*** Using a highly specific mass-spectrometry assay, we found no evidence of elevated circulating lipopolysaccharide in severe community-acquired pneumonia, including COVID-19. These findings challenge the concept that gut-derived endotoxemia is a major driver of systemic inflammation in severe pneumonia.

***Tweet*** Mass spectrometry reveals no rise in plasma LPS in severe pneumonia or COVID-19, questioning gut endotoxemia’s role in inflammation.

**Supplementary Information:**

The online version contains supplementary material available at 10.1186/s40635-026-00863-y.

## Background

Acute community-acquired pneumonia (CAP) remains the leading cause of death from infectious diseases worldwide. The most frequent pathogens are viruses, including SARS-CoV-2, influenza viruses, and Gram-positive bacteria, primarily *Streptococcus pneumoniae*. Severe cases are marked by a dysregulated host immune response, which contributes to circulatory failure, multi-organ dysfunction, and thrombotic complications [1].

Endotoxemia**,** defined by elevated plasma concentrations of lipopolysaccharide (LPS), is a central driver of inflammation and thrombosis in Gram-negative bacterial sepsis [1]. However, preclinical and clinical evidence suggests that LPS translocation into the bloodstream may also occur in early-stage sepsis of *non*-Gram-negative origin and is associated with poor outcomes [2–4]. In patients with CAP, circulating LPS levels can be comparable to levels observed in Gram-negative bacteremia, despite the absence of LPS-expressing pathogens [5]. In addition, low-grade endotoxemia is associated with short- and long-term risk of cardiovascular events in CAP patients [6]. Low-grade endotoxemia has also been detected in COVID-19 [7–10]. Interestingly, the SARS-CoV-2 spike protein may bind bacterial LPS and enhance its pro-inflammatory effects [11], and could favor thrombosis by eliciting a pro-inflammatory and pro-coagulant state.

This suggests that endotoxemia may reflect gut-derived bacterial translocation, driven by intestinal barrier dysfunction during critical illness, regardless of the primary pathogen. Elevated LPS levels have been associated with features of sepsis-induced immunoparalysis and may also be exacerbated by mechanical ventilation, which in preclinical models promotes enterocyte apoptosis and intestinal barrier breakdown [12].

Digestive decontamination with non-absorbable antibiotics has been shown in meta-analyses to reduce multi-organ dysfunction in ICU patients [13]. Additional non-antibiotic strategies are under investigation. Our group showed that recombinant phospholipid transfer protein (PLTP), a plasma protein that binds and neutralizes LPS, improves outcomes in murine models of peritonitis [14].

Overall, endotoxemia may serve as a biomarker of intestinal injury and systemic severity in severe pneumonia, independently of microbial etiology. A better understanding of its role could open new therapeutic avenues, including LPS neutralization or removal strategies.

However, the immunoassays currently used for LPS quantification suffer from technical limitations, including poor sensitivity and cross-reactivity, particularly with immunoassay-based tests, which call into question the reliability of the results obtained using these methods.

Our study aims to compare plasma concentrations of LPS between patients with severe community-acquired pneumonia (CAP) caused by SARS-CoV-2 (sCOVID-19) or other pathogens (sCAP) and healthy volunteers (HV), using a patented high-performance liquid chromatography–tandem mass spectrometry (LC–MS/MS) method that detects 3-hydroxy fatty acids (C10, C12, C14, C16, and C18), components of the lipid A moiety of LPS. Secondary analyses explored associations between LPS levels, immune and coagulation markers, gut translocation, and clinical outcomes.

## Methods

This study is a prospective, exploratory ancillary analysis of the ongoing LYMPHONIE project (ClinicalTrials.gov identifier: NCT03505281), initiated in November 2018 at the Dijon-Bourgogne University Hospital (France).

### Patients with severe community-acquired pneumonia

Eligible patients were those with severe community-acquired pneumonia, defined by the presence of: (1) clinical and radiological signs of pneumonia (at least two acute symptoms such as cough, purulent sputum, dyspnea, chest pain, body temperature < 35 °C or ≥ 38.5 °C, along with a new pulmonary infiltrate on imaging); (2) severity criteria, including at least two quick-SOFA components (systolic blood pressure ≤ 100 mmHg, respiratory rate ≥ 22 breaths/min, or Glasgow Coma Scale < 15), and/or the need for mechanical ventilation and/or vasopressors; and (3) diagnosis made within 48 h of hospital admission.

Non-inclusion criteria included age under 18 years, pregnancy, legal protection status, decisions to limit care, known immunodeficiency, chronic conditions associated with profound lymphopenia (e.g., cirrhosis, lymphoproliferative or myeloproliferative disorders, solid malignancy, or active systemic lupus erythematosus), or hospitalization for sepsis within the previous 3 months.

Patients with sCOVID-19 were included if they tested positive for SARS-CoV-2 by reverse transcriptase-polymerase chain reaction (RT-PCR) on a respiratory sample, whereas severe community-acquired pneumonia (sCAP) patients were those with no positive test for COVID-19.

Oral informed consent was obtained from patients or their legal representatives. The study was approved by the regional ethics committee (Comité de Protection des Personnes SUD MEDITERRANEE V; reference 2017-A03404-49). Blood samples collected in ethylenediaminetetraacetic acid (EDTA) tubes were drawn within 48 h of admission. After centrifugation at 2000 × g for 10 min at 4 °C, plasma was aliquoted and stored at − 80 °C in the certified biological resource center (CRB Ferdinand Cabanne; NF S96-900 certified).

### Healthy volunteers

We prospectively enrolled 34 healthy volunteers (HV). Inclusion criteria were: (i) age ≥ 18 years with oral consent provided; (ii) no fever, antibiotic use, or surgery within the previous 30 days; (iii) body temperature < 37.8 °C on the day of inclusion and no signs of suspected infection; and (iv) absence of any known immune deficiency. EDTA-treated whole blood was collected, and plasma was subsequently stored under the same conditions as for patient samples.

To ensure data comparability, we performed two separate matching procedures: one between HV and with sCOVID-19 patients, and the other between HV and sCAP patients. In both cases, matching was performed on sex and age. No matching was applied for comorbidities or body mass index.

### Variables of interest, clinical outcomes, and data collection

Clinical and biological parameters, along with the Charlson Comorbidity Index and severity scores—including the Sequential Organ Failure Assessment (SOFA), Simplified Acute Physiology Score II (SAPS II), and Pneumonia Severity Index (PSI)—were recorded at the time of inclusion. Acute respiratory distress syndrome (ARDS) was defined according to the Berlin criteria [15], while septic shock was defined as persistent hypotension requiring vasopressor support with a serum lactate concentration > 2 mmol/l despite adequate fluid resuscitation.

Clinical outcomes were assessed up to 30 days following hospital admission. These included 30-day mortality, the number of hospital-, ICU-, and ventilator-free days, as well as the occurrence of venous thromboembolic events and cardiovascular events (CVE).

Ventilator-free days were defined as the number of days alive between day 1 and day 30 after the onset of severe pneumonia during which the patient was breathing without mechanical ventilation.

Venous thromboembolic events were diagnosed during routine care, using computed tomography pulmonary angiography for pulmonary embolism and vascular ultrasonography for deep vein thrombosis. Cardiovascular events included myocardial infarction, ischemic stroke, or cardiovascular death.

All data were collected by trained clinical research assistants using a standardized electronic case report form (Cleanweb™), which included automated validation rules to flag missing or inconsistent entries. In addition, all data underwent double-checking to ensure accuracy and completeness.

### Plasma biomarkers of immune and coagulopathy response, and gut dysfunction

Fourteen plasma analytes were quantified using a Luminex multiplex assay (R&D Systems, USA), following the manufacturer’s instructions. These included cytokines and chemokines [TNF, IL-1β, IL-6, IL-8, IL-10, chemokine (C–C motif) ligand 2 (CCL2), and chemokine (C-X-C motif) ligand 10 (CXCL10)]; markers of monocyte/macrophage activation [soluble CD14 (sCD14) and soluble CD163 (sCD163)]; and biomarkers of coagulopathy [D-dimer, tissue factor, thrombomodulin, antithrombin III, and soluble vascular cell adhesion molecule-1 (sVCAM-1)]. All measurements were performed on the same day by the same operator to minimize inter-assay variability.

In addition, human plasma concentrations of intestinal fatty acid-binding protein (I-FABP), a surrogate marker of gut barrier integrity, were measured using a commercially available enzyme-linked immunosorbent assay (ELISA), also following the manufacturer’s protocol (R&D Systems, Minneapolis, MN, USA).

### Lipopolysaccharide measurement

Plasma LPS concentrations were measured using a patented LC–MS/MS method (EndoQuant), specifically designed for the targeted quantification of 3-hydroxy (3-OH) fatty acids (C10, C12, C14, C16, and C18), which are covalently linked to the lipid A moiety of LPS [16]. For this purpose, two plasma aliquots (100 µL each) were spiked with 4 pmol of internal standards (3-hydroxyundecanoic, 3-hydroxytridecanoic, 3-hydroxyheptadecanoic).

Plasma was hydrolyzed (for total 3-OH fatty acids) or left unhydrolyzed (for unesterified 3-OH fatty acids) using 8 M HCl for 3 h at 90 °C. Fatty acids were then extracted using a hexane/ethyl acetate (3:2) solvent mixture and reconstituted in ethanol after vacuum evaporation. Chromatographic separation was done using an Infinity 1260 HPLC binary system (Agilent) equipped with a ZORBAX SB-C18 column (50 × 2.1 mm, 1.8 µm; Agilent) maintained at 30 °C. MS/MS detection was carried out on an Agilent QQQ 6490 triple quadrupole mass spectrometer fitted with a Jet-Stream electrospray ionization (ESI) source. Quantification of 3-OH fatty acids was achieved in negative selected reaction monitoring (SRM) mode, as previously described [16, 17].

The esterified fraction of 3-OH fatty acids (i.e., those bound to LPS) was calculated by subtracting the concentration of unesterified 3-OH fatty acids from the total concentrations. Final plasma LPS concentrations were expressed as the sum of esterified 3-OH fatty acids.

### Statistical analyses

Categorical variables were summarized as counts and percentages, and continuous variables were described using medians and interquartile ranges (IQR), as most variables did not follow a normal distribution. Baseline patient (sCOVID-19 and sCAP) and HV characteristics were described and compared between groups. The percentage of patients with low and high plasma LPS levels, defined according to the median value of LPS in patients (sCAP and sCOVID-19), was described and compared. Using the median plasma LPS concentration as the cutoff is an unbiased data-driven approach chosen in the absence of an established clinical or biological threshold and to ensure balanced group sizes. Categorical variables were compared using Fisher’s exact test or McNemar’s test, as appropriate. For continuous variables, the Wilcoxon signed-rank test was used. Spearman’s correlation coefficients were calculated to assess correlations, as appropriate. *P*-values < 0.05 were considered statistically significant and are highlighted in bold in the tables. Statistical analyses were conducted using R software (version 4.3.0), and figures were generated with GraphPad Prism (version 10.0.3).

## Results

### Population included

We included 34 patients with sCAP, 34 with sCOVID-19, and 34 matched HV. Among patients with sCAP, the etiologies were bacterial in 11 patients (32%), viral in 8 (24%), mixed in 5 (15%), and unknown in 10 (29%). In the sCOVID-19 group, 4 patients (12%) had a documented bacterial coinfection (Suppl. Table 1).

Intensive care unit admission was required for 31 (91%) of sCAP patients and 34 (100%) of sCOVID-19 patients. Median SOFA score was 9.5 (6-11) for sCAP patients, and 7 (4-8) for sCOVID-19 patients. Septic shock and ARDS were reported in 10 (29%) and 21 (62%) of sCAP patients, and in 4 (12%) and 31 (91%) of sCOVID-19 patients, respectively. Two-thirds of patients required vasopressors. Venous thrombosis was reported in 4 (12%) of sCAP patients, and 6 (18%) of sCOVID-19 patients. Cardiovascular events were reported in 6 (18%) of patients from both the sCAP and sCOVID-19 groups. Thirty-day mortality was 6% and 12% for sCAP patients and sCOVID-19 patients, respectively (Table 1).

**Table 1:** Baseline characteristics and outcomes of the study population (LYMPHONIE study, 2018–2022)

	Normal range	Missing data*	Study group			*P* value	
			Healthy	sCAP	sCOVID-19	H versus NC	H versus C
			*N* = 34	*N* = 34	*N* = 34		
*Demographics*							
Age (yr), median (IQR)			63 (52–71)	66 (57–71)	65 (52–71)	0.107	0.130
Male sex, *n* (%)			20 (59)	26 (76)	19 (56)	0.077	0.999
Body-mass index (kg/m^2^), median (IQR)			25 (24–29)	29 (27–37)	31 (26–35)	**0.003**	**0.004**
*Chronic comorbidities*							
Cardiovascular disease, *n* (%)			2 (6)	8 (24)	6 (18)	**0.041**	0.289
Pulmonary disease, *n* (%)			0	12 (35)	4 (12)	**0.001**	0.134
Diabetes mellitus, *n* (%)			1 (3)	10 (29)	12 (35)	**0.016**	**0.003**
Charlson score			0 (0–1)	1 (0–2)	1 (0–1)	**0.001**	0.074
*Severity at hospital admission*							
Septic shock, *n* (%)			0	10 (29)	4 (12)		
ARDS, *n* (%)			0	21 (62)	31 (91)		
Pneumonia severity index, median (IQR)			–	117 (86–135)	83 (71–111)		
SAPS II, median (IQR)			–	27 (17–35)	17 (9–26)		
SOFA score, median (IQR)			–	9.5 (6–11)	7 (4–8)		
*Biological findings on admission*							
ASAT (IU/l), median (IQR)	15–37	0/0/2	–	47 (27–97)	76 (45–111)		
Serum creatinine (μmol/l), median (IQR)	59–104		–	109 (73–172)	80 (61–100)		
NT-ProBNP (pg/ml), median (IQR)	< 125	0/0/1	–	1748 (640–7488)	283 (81–1322)		
PaO_2_:FiO_2_ (mm Hg), median (IQR)	≥ 400		–	118 (72–166)	115 (86–172)		
Arterial pH (mm Hg), median (IQR)	7.35–7.45		–	7.37 (7.24–7.43)	7.44 (7.35–7.46)		
Serum bicarbonate (mmol/l), median (IQR)	20–29	0/0/1	–	23.5 (21–27)	25 (23–27)		
Lactate level (mmol/l), median (IQR)	0.5–2.0		–	2.15 (1.40–3.60)	1.50 (1.30–2.50)		
C-reactive protein (mg/l), median (IQR)	< 3.2	0/0/2	2 (2–2)	220 (107–417)	174 (137–224)	** < 0.001**	** < 0.001**
Platelets (× 10^9^/l), median (IQR)	150–400		232 (198–268)	171.5 (144–244)	240 (176–318)	** < 0.013**	0.432
Leukocytes (× 10^6^/l), median (IQR)	3.8–9.5		6.3 (5.2–7.3)	10.4 (7.5–14.2)	7.9 (4.7–12.8)	** < 0.001**	**0.002**
Neutrophils (× 10^6^/l), median (IQR)	1.7–5.8		3.5 (2.7–4.3)	9.1 (6.6–14.7)	6.5 (3.7–11.2)	** < 0.001**	** < 0.001**
Lymphocytes (× 10^3^/l), median (IQR)	1.07–4.03		1625 (1325–2175)	545 (290–900)	755 (530–820)	** < 0.001**	** < 0.001**
Monocytes (× 10^3^/l), median (IQR)	0.2–0.7		420 (345–595)	415 (220–860)	315 (220–540)	0.681	0.094
*Treatments*							
ICU admission, *n* (%)			–	31 (91)	34 (100)		
Antibiotic multitherapy, *n* (%)			–	26 (76)	19 (56)		
Corticosteroids, *n* (%)			–	20 (59)	24 (71)		
Invasive mechanical ventilation, *n* (%)			–	25 (74)	27 (79)		
Renal replacement therapy, *n* (%)			–	7 (21)	2 (6)		
Vasopressors, *n* (%)			–	20 (59)	19 (56)		
*Outcomes*							
Venous thrombosis event, *n* (%)			–	4 (12)	6 (18)		
Cardiovascular event, *n* (%)				6 (18)	6 (18)		
ICU-free days at 30 days (days), median (IQR)			–	18 (12–27)	16 (10–30)		
Ventilator-free days at 30 days (days), median (IQR)			–	24 (14–30)	16 (10–30)		
Hospital-free days at 30 days (days), median (IQR)			–	4.5 (0–16)	0.50 (0–12)		
30-day mortality, *n* (%)			–	2 (6)	4 (12)		
90-day mortality, *n* (%)			–	2 (6)	5 (15)		

ARDS, acute respiratory distress syndrome; ICU, intensive care unit; IQR, interquartile range; sCAP, severe community-acquired pneumonia

*Missing data: the notation *X*/*Y*/*Z* now indicates the number of missing values in the three respective groups (healthy volunteers/sCAP/sCOVID-19)

### Comparison of characteristics and LPS levels in sCAP and sCOVID-19 patients with matched healthy volunteers

As expected, median age and sex ratio did not differ significantly between the patient groups and HV. However, sCAP patients had significantly higher body mass index (BMI), more frequent cardiovascular and pulmonary comorbidities, diabetes, and a higher Charlson comorbidity index. Compared to HV, sCOVID-19 patients also had higher BMI and a greater prevalence of diabetes (Table 1).

**Fig. 1:**
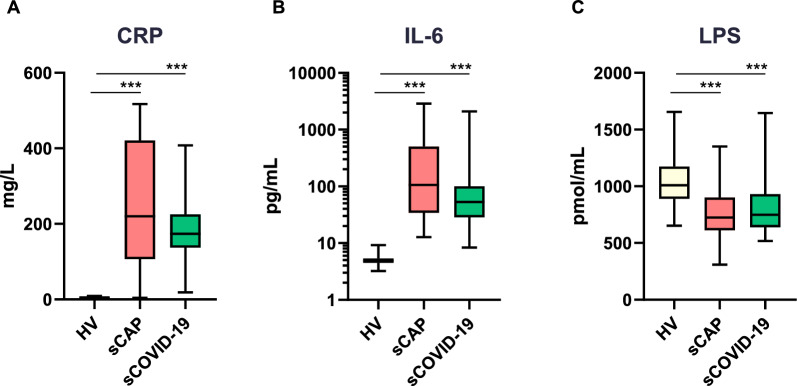
Plasma LPS concentrations and inflammatory response in healthy volunteers and patients with severe community-acquired or COVID-19 pneumonia (LYMPHONIE study, 2018–2022). Data are presented as box-and-whisker plots and differences were analyzed using the Wilcoxon matched-pairs signed rank test and significance indicated as *p < 0.05; **p < 0.01; ***p < 0.001; ****p < 0.0001

Compared to HV, patients with sCAP and sCOVID-19 exhibited significantly elevated levels of inflammatory biomarkers (CRP, TNF, IL-1β, IL-6, IL-8, CCL2, CXCL10, sCD14) as well as markers of coagulopathy (D-dimer, sVCAM-1), but showed lower levels of soluble I-FABP (Table 2, Fig. 1).

**Table 2:** Plasma concentration of biomarkers of immune and coagulopathy responses, gut dysfunction, and LPS (LYMPHONIE study, 2018–2022)

	Study group			*P* value	
	Healthy	sCAP	sCOVID-19	H versus NC	H versus C
	*N* = 34	*N* = 34	*N* = 34		
*Cytokines, chemokines*					
TNF (pg/ml)	7 (6–8)	16 (10–20)	12 (11–15)	** < 0.001**	** < 0.001**
IL-1β (pg/ml)	12 (10–13)	17 (13–21)	13 (12–15)	** < 0.001**	**0.006**
IL-6 (pg/ml)	5 (4–5)	106 (36–480)	53 (29–100)	** < 0.001**	** < 0.001**
IL-8 (pg/ml)	2 (2–2)	6 (3–19)	6 (4–10)	** < 0.001**	** < 0.001**
IL-10 (pg/ml)	8 (4–11)	8 (6–13)	8 (6–10)	0.064	0.385
CCL2 (pg/ml)	181 (160–212)	315 (189–680)	342 (248–528)	** < 0.001**	** < 0.001**
CXCL10 (pg/ml)	35 (27–44)	231 (77–662)	492 (323–692)	** < 0.001**	** < 0.001**
*Biomarkers of monocyte/macrophage activation*					
sCD14 (ng/ml)	2243 (1863–2601)	3068 (2623–3542)	3714 (2928–4080)	** < 0.001**	** < 0.001**
sCD163 (ng/ml)	594 (450–814)	751 (515–1031)	636 (547–864)	0.057	**0.033**
*Biomarkers of coagulopathy*					
D-dimer (ng/ml)	1114 (641–2037)	9860 (4337–16,501)	5901 (3665–8581)	** < 0.001**	** < 0.001**
Tissue-factor (pg/ml)	39 (35–49)	39 (32–54)	55 (47–66)	0.343	** < 0.001**
Thrombomodulin (ng/ml)	5.43 (4.48–6.12)	6.58 (4.39–8.67)	5.55 (4.59–7.53)	**0.018**	0.163
Antithrombin-III (ng/ml)	3775 (2900–4384)	2470 (2156–3284)	4107 (3219–4639)	** < 0.001**	0.134
sVCAM-1 (ng/ml)	742 (663–1008)	2258 (1376–3419)	4241 (2814–5188)	** < 0.001**	** < 0.001**
*Biomarker of gut dysfunction*					
sI-FABP (pg/ml)	362 (242–560)	240 (111–394)	173 (96–263)	0.078	**0.008**
*Endotoxemia measurement*					
LPS (pmol/ml)	1009 (921–1163)	724 (615–872)	750 (640–930)	** < 0.001**	** < 0.001**

Median and interquartile range are represented

CCL, chemokine (C–C motif) ligand; CXCL, chemokine (C-X-C motif) ligand; IL, interleukin; LPS, lipopolysaccharide; PCT, procalcitonin; sVCAM, soluble vascular cell adhesion molecule-1; sI-FABP, soluble intestinal fatty acid-binding protein; TNF, tumor necrosis factor

Unexpectedly, median plasma concentrations of LPS were significantly lower in both sCAP patients (724 [615–872] pmol/ml) and sCOVID-19 patients (750 [640–930] pmol/ml), compared to matched HV (1009 [921–1163] pmol/ml; *p* < 0.001 for both comparisons) (Table 2, Fig. 1).

To investigate potential factors that could explain this difference, we observed a significant decrease in hemoglobin levels in both sCAP and sCOVID-19 patients, as well as a significant reduction in hematocrit in sCAP, compared with healthy volunteers, suggesting the presence of hemodilution (Supplemental Fig. S1).

### Variables associated with high LPS levels

Patients with severe pneumonia (sCAP and sCOVID-19) were stratified into low and high LPS groups based on the median plasma LPS concentrations. Patients in the high LPS group more frequently had underlying pulmonary disease (*p* = 0.015) and tended to have a higher frequency of cardiovascular comorbidities (30% vs. 11%; *p* = 0.054). However, no significant differences were observed between the two groups in terms of septic shock or ARDS occurrence, SOFA scores, or levels of inflammatory and coagulopathy biomarkers. The proportion of pneumonia cases caused by Gram-negative bacteria was similar in both groups. Likewise, clinical outcomes did not differ significantly between the two groups (Tables 3 and 4).

**Table 3:** Baseline characteristics and outcomes according to LPS plasma concentrations (LYMPHONIE study, 2018–2022)

	Missing data*	Overall	Low LPS	High LPS	*P* value
		*N* = 68	*N* = 35	*N* = 33	
*Demographics*					
Age (yr), median (IQR)		65 (57–71)	65 (54–71)	65 (57–71)	0.893
Male sex, *n* (%)		45 (66)	22 (63)	23 (70)	0.551
Body-mass index (kg/m^2^), median (IQR)		30.6 (26.4–36.8)	30.8 (26.3–37.2)	30.4 (26.5–36.4)	0.961
*Chronic comorbidities*					
Cardiovascular disease, *n* (%)		14 (21)	4 (11)	10 (30)	0.054
Pulmonary disease, *n* (%)		16 (24)	4 (11)	12 (36)	**0.015**
Diabetes mellitus, *n* (%)		22 (32)	12 (34)	10 (30)	0.726
Charlson score		1 (0–1)	1 (0–1)	1 (0–1)	0.534
*Severity at hospital admission*					
Septic shock, *n* (%)		14 (21)	7 (20)	7 (21)	0.902
ARDS, *n* (%)		52 (76)	27 (77)	25 (76)	0.893
Pneumonia severity index, median (IQR)		93 (73–128.50)	91 (72–131)	105 (77–128)	0.641
SAPS II, median (IQR)		22 (14.50–34)	22 (17–30)	21 (11–36)	0.888
SOFA score, median (IQR)		7.5 (4.5–10)	8 (5–10)	7 (4–10)	0.980
*Biological findings on admission*					
ASAT (IU/l), median (IQR)	0/2	56.50 (38–110)	56 (37–115)	67 (40–97)	0.782
Serum creatinine (μmol/l), median (IQR)		87 (63.50–135)	78 (59–136)	90 (79–117)	0.116
NT-ProBNP (pg/ml), median (IQR)	0/1	946 (229–4111)	816 (194–1712)	1497 (259–9207)	0.169
PaO_2_:FiO_2_ (mm Hg), median (IQR)		115 (80–166)	109 (79–163)	141 (85–166)	0.439
Arterial pH (mm Hg), median (IQR)		7.40 (7.30–7.45)	7.40 (7.30–7.45)	7.40 (7.30–7.45)	0.610
Serum bicarbonate (mmol/l), median (IQR)	0/1	24 (21–27)	25 (21–27)	23.50 (21.50–26)	0.416
Lactate level (mmol/l), median (IQR)		1.80 (1.30–2.80)	1.80 (1.10–2.50)	2.10 (1.40–3.39)	0.087
C-reactive protein (mg/l), median (IQR)	1/1	186.70 (129.20–301)	186.70 (113–301)	182.50 (132.60–296.75)	0.677
Platelets (× 10^9^/l), median (IQR)		205.50 (157–294.5)	211 (163–344)	197 (146–265)	0.162
Leukocytes (× 10^6^/l), median (IQR)		8.9 (6.6–13.8)	9.1 (6.7–14.2)	8.5 (5.6–13.4)	0.544
Neutrophils (× 10^6^/l), median (IQR)		7.9 (5.3–13.1)	8.0 (6.1–13.9)	7.6 (4.8–12.4)	0.354
Lymphocytes (× 10^3^/l), median (IQR)		665 (390–860)	760 (530–900)	560 (290–830)	0.082
Monocytes (× 10^3^/l), median (IQR)		385 (220–685)	420 (230–670)	310 (190–700)	0.361
*Microbiological features*					
Bacterial etiology identified, *n* (%)		20 (29)	9 (26)	11 (33)	0.491
Gram-negative bacteria identified, *n* (%)		15 (22)	8 (23)	7 (21)	0.870
*Treatments*					
ICU admission, *n* (%)		65 (96)	34 (97)	31 (94)	0.608
Antibiotic multitherapy, *n* (%)		45 (66)	26 (74)	19 (58)	0.145
Corticosteroids, *n* (%)		44 (65)	23 (66)	21 (64)	0.858
Invasive mechanical ventilation, *n* (%)		52 (76)	28 (80)	24 (73)	0.480
Renal replacement therapy, *n* (%)		9 (13)	3 (9)	6 (18)	0.299
Vasopressors, *n* (%)		39 (57)	23 (66)	16 (48)	0.151
*Outcomes*					
Venous thrombosis event, *n* (%)		10 (15)	5 (14)	5 (15)	0.999
Cardiovascular event, *n* (%)		12 (18)	8 (23)	4 (12)	0.246
ICU-free days at 30 days (days), median (IQR)		16.5 (9–26.5)	17 (12–28)	16 (6–25)	0.999
Ventilator-free days at 30 days (days), median (IQR)		22 (11.5–30)	24 (14–30)	21 (11–30)	0.168
Hospital-free days at 30 days (days), median (IQR)		1.5 (0–14.5)	6 (0–14)	0 (0–16)	0.761
30-day mortality, *n* (%)		6 (9)	2 (6)	4 (12)	0.421
90-day mortality, *n* (%)		7 (10)	3 (7)	4 (12)	0.705

ARDS, acute respiratory distress syndrome; ICU, intensive care unit; IQR, interquartile range; sCAP, severe community-acquired pneumonia

*Missing data: the notation X/Y/Z now indicates the number of missing values in the two respective groups (low LPS/high LPS)

**Table 4:** Plasma concentration of biomarkers of immune and coagulopathy responses, gut dysfunction according to LPS plasma concentrations (LYMPHONIE study, 2018–2022)

	Overall	Low LPS	High LPS	*P* value
	*N* = 68	*N* = 35	*N* = 33	
*Cytokines, chemokines*				
TNF (pg/ml)	13 (10–18)	13 (10–17)	13 (11–20)	0.690
IL-1β (pg/ml)	15 (12–18)	14 (12–18)	15 (12–19)	0.424
IL-6 (pg/ml)	64 (30–158)	73 (33–159)	59 (29–158)	0.820
IL-8 (pg/ml)	6 (4–13)	5 (3–13)	7 (4–14)	0.244
IL-10 (pg/ml)	8 (6–11)	7 (6–11)	8 (6–13)	0.454
CCL2 (pg/ml)	324 (208–572)	309 (248–582)	357.22 (189–563)	0.927
CXCL10 (pg/ml)	412 (170–677)	360 (211–733)	423 (142–611)	0.615
*Biomarkers of monocyte/macrophage activation*				
sCD14 (ng/ml)	3310 (2853–3988)	3239 (2910–3661)	3542 (2781–4080)	0.331
sCD163 (ng/ml)	720 (537–1009)	663 (547–864)	775 (535–1023)	0.615
*Biomarkers of coagulopathy*				
D-dimer (ng/ml)	6849 (4004–13046)	5482 (3684–11,658)	7916.81 (5382–13,159)	0.163
PCT (μg/l)	1.24 (0.22–13.70)	0.77 (0.22–12)	2.81 (0.29–16.45)	0.517
Tissue-factor (pg/ml)	49.2 (36.7–63.5)	51.5 (36.7–64.4)	48.6 (36.7–62.6)	0.830
Thrombomodulin (ng/ml)	6.1 (4.6–8.2)	6.4 (4.5–7.5)	5.9 (4.8–8.5)	0.480
Antithrombin-III (ng/ml)	3281 (2470–4147)	2871 (2288–4225)	3472 (2660–4140)	0.239
sVCAM-1 (ng/ml)	2944 (2005–4747)	2718 (1889–4755)	3560 (2469–4739)	0.227
*Biomarker of gut dysfunction*				
sI-FABP (pg/ml)	183 (107–380)	178 (89–394)	184 (120–378)	0.641

Median and interquartile range are represented

CCL, chemokine (C–C motif) ligand; CXCL, chemokine (C-X-C motif) ligand; IL, interleukin; LPS, lipopolysaccharide; PCT, procalcitonin; sVCAM, soluble vascular cell adhesion molecule-1; sI-FABP, soluble intestinal fatty acid-binding protein; TNF, tumor necrosis factor

No significant correlation was observed between LPS levels and severity scores (SOFA, PSI, or SAPS II) at day 0.

**Fig. 2:**
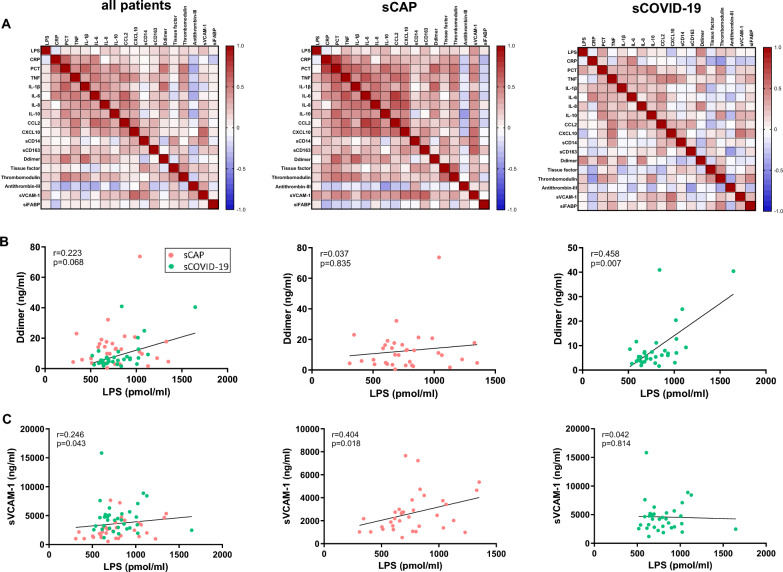
Correlations between plasma LPS concentrations and immune or coagulopathy biomarkers (LYMPHONIE study, 2018–2022)

Most correlations between plasma LPS levels and biomarkers of immune or coagulopathy were weak and not statistically significant, suggesting little to no association. However, a low correlation was observed between LPS and soluble VCAM-1 (sVCAM-1) in the overall population (*r* = 0.246, *p* = 0.043), with a stronger correlation in sCAP patients (*r* = 0.404, *p* = 0.018). Similarly, LPS levels and D-dimer levels showed a low correlation with the overall population (*r* = 0.223, *p* = 0.043), and a more pronounced correlation in patients with sCOVID-19 (*r* = 0.458, *p* = 0.007) (Fig. 2).

## Discussion

In this prospective study using a validated LC–MS/MS-based quantification method, we observed that patients with severe pneumonia, including COVID-19 and CAP resulting from other etiologies, did not have increased plasma concentrations of LPS compared to matched healthy volunteers. These findings challenge previous assumptions and reports suggesting elevated endotoxemia in severe pneumonia and sepsis regardless of microbial etiology.

Following earlier studies reporting higher LPS levels in CAP patients, it was suggested that endotoxemia results from gut barrier dysfunction and microbial translocation, even in infections caused by non-Gram-negative pathogens. This “intestinal hypothesis” has gained traction as disruption of the digestive barrier is a well-recognized consequence of critical illness, and low-grade endotoxemia has been associated with adverse outcomes, including cardiovascular events [3, 6]. However, many of these findings were based on LAL (Limulus Amebocyte Lysate) assays that predominantly reflect the biologically active portion of circulating LPS, rather than its total burden. In addition, LAL-based assays are prone to false positives due to cross-reactivity with plasma components such as (1- > 3)-β-d-glucans and proteases [18, 19]. Moreover, plasma is a complex biological matrix containing lipoproteins and other factors capable of binding or inactivating LPS [16]. By contrast, our use of mass spectrometry to measure esterified 3-hydroxy fatty acids, specific components of lipid A, enables direct, sensitive, and specific quantification of total LPS mass [16, 17, 20]. This robust methodology may explain the discrepancy with prior studies and underscores the importance of assay selection when evaluating circulating LPS. Then, alterations in lipid metabolism during acute inflammation may reduce measurable plasma LPS levels through enhanced binding to LDL and VLDL particles and subsequent hepatic clearance, while tissue sequestration of LPS during severe organ dysfunction may further limit its circulation [21–26]. In addition, hemodilution related to fluid resuscitation likely contributes to lower plasma LPS concentrations in pneumonia patients, as supported by reduced hemoglobin and hematocrit levels. Differences in gut microbiota composition, potentially influenced by early antibiotic administration in the emergency department, cannot be excluded, although selective digestive tract decontamination was not used in our cohort.

Interestingly, despite lower LPS levels in patients compared to healthy controls, we observed a low positive correlation between LPS and markers of endothelial activation (sVCAM-1) and coagulation (D-dimer), particularly within each pneumonia subgroup. These findings suggest that even relatively low concentrations of circulating LPS might contribute to endothelial dysfunction and pro-thrombotic states, which are prominent features of severe pneumonia, including COVID-19. However, no significant association was found between LPS levels and the incidence of venous thromboembolism or cardiovascular events in our cohort, unlike previous studies [6].

Contrary to our expectations, plasma concentrations of I-FABP, a marker of enterocyte injury and gut permeability, were significantly lower in patients than in healthy volunteers. Similar results were observed in other studies of pneumonia and COVID-19 [27–29], and may reflect altered enterocyte turnover or plasma protein dilution during critical illness rather than preserved gut integrity. This finding, along with low LPS levels, does not support the hypothesis of increased gut-derived LPS translocation in our patient cohort.

Moreover, high LPS levels were not associated with more severe disease features such as septic shock, ARDS, or higher SOFA scores. The prevalence of Gram-negative pathogens was similar in patients with high vs. low LPS concentrations, further arguing against a direct microbial origin of circulating LPS.

Our findings align with prior experimental and clinical results showing the absence of significant endotoxemia during pneumococcal pneumonia, even in the presence of systemic inflammation and organ failure [29]. Together, these results challenge the concept of LPS translocation as a major driver of systemic inflammation in CAP and COVID-19.

The strengths of our study include its prospective design, use of a validated and highly specific LC–MS/MS assay for LPS quantification, and inclusion of well-characterized patient cohorts with matching to healthy controls. Biomarker measurements were centralized and standardized, reducing variability.

However, several limitations should be acknowledged. First, the sample size was relatively small, limiting statistical power for subgroup analyses. This is particularly relevant for rare outcomes such as thromboembolic or cardiovascular events. Second, despite matching, residual confounding due to comorbidities or medications cannot be entirely excluded. Then, patients and HV differed in comorbid conditions, introducing potential confounders in the interpretation of plasma LPS concentrations. Indeed, we only matched patients and HV on age and sex. However, this should be regarded as a conservative bias since certain comorbidities (i.e., chronic respiratory and cardiovascular or renal disease and high BMI) have been previously associated with increased endotoxemia and/or enhanced intestinal permeability [30–35]. Furthermore, we cannot exclude the possibility of later increases in LPS concentrations, as our samples were collected early (< 48 h). The findings of Kritselis et al. suggest that LPS kinetics may be ascending in some patients, especially those with sepsis secondary to pneumonia who ultimately do not survive compared to survivors [5]. Importantly, given the limited sample size, these results should be interpreted with caution. Lastly, I-FABP may be an imperfect marker of gut permeability in severe pneumonia. Conditions such as systemic inflammation, metabolic disturbances, and multi-organ dysfunction can alter enterocyte function and protein release, thereby reducing I-FABP levels in the circulation independently of overt epithelial injury [28, 36, 37]. However, we cannot rule out a potential effect of hemodilution to explain the lower concentrations of I-FABP in patients compared to HV.

## Conclusions

In conclusion, using a highly specific LC–MS/MS assay, we found no evidence of increased plasma LPS concentrations in patients with sCOVID-19 or sCAP compared to HV. These findings suggest that gut-derived endotoxemia does not play a major role in the pathophysiology of severe pneumonia. Although moderate correlations between LPS and markers of endothelial activation were observed, LPS levels were not associated with clinical severity or outcomes in our series. Future studies with larger, well-stratified cohorts are needed to evaluate the activity and functional consequences of circulating LPS, and to better define the role of gut–lung crosstalk in pneumonia-related inflammation and thrombosis.

## Supplementary Information

Additional file 1.

## Data Availability

The datasets analyzed during the current study are available from the corresponding author on reasonable request.

## Abbreviations

BALF: Bronchoalveolar lavage fluid
CRP: C-reactive protein
I-FABP: Intestinal fatty acid-binding protein
IL-6: Interleukin 6
LC–MS/MS: Liquid chromatography coupled to tandem mass spectrometry
LPS: Lipopolysaccharide
sCAP: Severe community-acquired pneumonia
sCOVID-19: Severe COronaVIrus Disease 2019
