# Supplementary material for: Endotoxemia and its association with immune and coagulopathy responses in severe community-acquired pneumonia and COVID-19
Source: Intensive Care Med Exp. 2026 Mar 9;14:31. doi: 10.1186/s40635-026-00863-y (PMC12968107; doi:10.1186/s40635-026-00863-y)
Supplement: Supplementary file 1 — Additional file 1. [file 40635_2026_863_MOESM1_ESM.docx]

**Supplemental table 1 :** microbiological etiology of pneumonia

|  | **Overall** | **sCAP** | **sCOVID-19** |
| --- | --- | --- | --- |
|  | **N=68** | **N=34** | **N=34** |
| **Pure Viral** | **38 (56%)** | **8 (24%)** | **30 (88%)** |
| Influenza A |  | 6 (18%) | 0 |
| Rhinovirus |  | 1 | 0 |
| RSV |  | 1 | 0 |
| SARS-CoV-2 |  | 0 | 30 (88%) |
| **Pure Bacterial** | **11 (16%)** | **11 (32%)** | **0** |
| *Streptococcus pyogenes* |  | 1 | 0 |
| *Streptococcus pneumoniae* |  | 1 | 0 |
| *Klebsiella pneumoniae* |  | 2 | 0 |
| *Haemophilus parainfluenzae* |  | 1 | 0 |
| *Pseudomonas aeruginosa* |  | 1 | 0 |
| *Morganella morganii + Proteus mirabilis* |  | 1 | 0 |
| *Mycoplasma pneumoniae* |  | 1 | 0 |
| *Legionella pneumophila* |  | 3 | 0 |
| **Mixed (Bacterial + Viral)** | **9 (13%)** | **5 (15%)** | **4 (12%)** |
| Influenza A + *Streptococcus pyogenes* |  | 1 |  |
| Influenza A + *Staphylococcus aureus + Streptococcus dysgalactiae* |  | 1 |  |
| Influenza A + *Staphylococcus aureus* |  | 1 |  |
| Metapneumovirus + *Haemophilus parainfluenzae* |  | 1 |  |
| Metapneumovirus + *Haemophilus influenzae + Staphylococcus aureus + Streptococus pneumoniae* |  | 1 |  |
| SARS-Cov-2 + *Staphylococcus aureus* |  |  | 1 |
| SARS-Cov-2 + *Enterococcus* |  |  | 2 |
| SARS-Cov-2 + *Enterobacter cloacae* |  |  | 1 |
| **Microbiologically undocumented pneumonia** | **10 (15%)** | **10 (29%)** | **0** |

**Supplemental table 2.** Spearman correlation between plasma LPS concentrations and biomarkers of immune and coagulopathy responses and gut dysfunction

|  | **Overall** | | **sPAC** | | **sCOVID-19** | |
| --- | --- | --- | --- | --- | --- | --- |
|  | **Correlation** | ***P* value** | **Correlation** | ***P* value** | **Correlation** | ***P* value** |
| **Routine inflammatory biomarkers** |  |  |  |  |  |  |
| Leukocytes | -0.10 | 0.422 | -0.10 | 0.581 | -0.14 | 0.423 |
| Neutrophils | -0.13 | 0.296 | -0.17 | 0.329 | -0.09 | 0.624 |
| Lymphocytes | -0.19 | 0.126 | -0.09 | 0.600 | -0.41 | **0.017** |
| Monocytes | -0.11 | 0.381 | -0.17 | 0.328 | 0.02 | 0.903 |
| C-reactive protein | -0.11 | 0.397 | -0.08 | 0.653 | -0.08 | 0.654 |
| Procalcitonin | 0.10 | 0.443 | 0.03 | 0.851 | 0.36 | **0.043** |
| **Cytokines, chemokines** |  |  |  |  |  |  |
| TNF | 0.10 | 0.395 | 0.26 | 0.139 | -0.03 | 0.857 |
| IL-1β | 0.09 | 0.456 | 0.14 | 0.425 | 0.13 | 0.456 |
| IL-6 | 0.02 | 0.896 | 0.08 | 0.640 | -0.02 | 0.915 |
| IL-8 | 0.19 | 0.125 | 0.20 | 0.255 | 0.22 | 0.209 |
| IL-10 | 0.19 | 0.125 | 0.28 | 0.110 | 0.08 | 0.642 |
| CCL2 | 0.05 | 0.672 | 0.06 | 0.736 | 0.07 | 0.674 |
| CXCL10 | 0.03 | 0.798 | 0.17 | 0.342 | -0.22 | 0.207 |
| **Biomarkers of monocyte/macrophage activation** | |  |  |  |  |  |
| sCD14 | 0.07 | 0.585 | 0.05 | 0.779 | 0.05 | 0.799 |
| sCD163 | 0.15 | 0.233 | 0.25 | 0.150 | -0.04 | 0.819 |
| **Biomarkers of coagulopathy** |  |  |  |  |  |  |
| D-dimer | 0.22 | **0.068** | 0.04 | 0.835 | 0.46 | **0.007** |
| Tissue-Factor | 0.07 | 0.593 | 0.18 | 0.321 | -0.10 | 0.584 |
| Thrombomodulin | 0.16 | 0.180 | 0.16 | 0.363 | 0.21 | 0.231 |
| Antithrombin-III | 0.11 | 0.377 | 0.06 | 0.738 | 0.18 | 0.305 |
| sVCAM-1 | 0.25 | **0.043** | 0.40 | **0.018** | 0.04 | 0.813 |
| **Biomarker of gut dysfunction** |  |  |  |  |  |  |
| I-FABP | 0.07 | 0.581 | 0.09 | 0.592 | 0.04 | 0.838 |

**Supplemental Figure 1.** Blood hemoglobin (A) and hematocrit (B) in healthy volunteers and patients with severe community-acquired or COVID-19 pneumonia (LYMPHONIE study, 2018–2022)

Data are presented as box-and-whisker plots and differences were analyzed using the Wilcoxon matched-pairs signed rank test and significance indicated as *, p < .05; **, p < .01; ***, p < .001; ****, p < .0001.

| **A**   | **B**   |
| --- | --- |
